# Acute interstitial nephritis during rifampicin therapy can be a paradoxical response: a case report

**DOI:** 10.1186/1757-1626-2-6643

**Published:** 2009-04-03

**Authors:** Jan van der Meulen, Gijs MT de Jong, Pieter J Westenend

**Affiliations:** 1 DIANET, Utrecht, The Netherlands; 2 Department of Nephrology, Albert Schweitzer Hospital, Dordrecht, The Netherlands; 3 Laboratory for Pathology, Dordrecht, The Netherlands

## Abstract

An 18-year-old Ethiopian woman presented with debilitating back pain and high fever. X-ray examinations showed diffuse pulmonary tuberculosis and a psoas abscess. After starting rifampicin, isoniazid, ethambutol and pyrazinamide, acute interstitial nephritis developed that spontaneously recovered. According to Harrison's Online rifampicin should have been causative, but the spontaneous recovery excluded that possibility. The clinical course fit the diagnosis of a paradoxical response, for which recently risk factors have been described. Thus, a paradoxical response should be added to the list of causes of interstitial nephritis in tuberculosis patients and in such cases rifampicin could be continued.

## Introduction

Rifampicin, mostly in combination with isoniazid, ethambutol and pyrazinamide, is the first-line therapy for tuberculosis. Rifampicin's most feared side effect is hepatotoxicity. However, nephrotoxicity such as acute tubular necrosis and interstitial nephritis (IN) have also been reported. In case of acute tubular necrosis, rifampicin-dependent antibodies have been found, suggesting a causal relationship between rifampicin and renal failure [1]. In acute IN only a temporal relationship is reported [2,4]. In spite of the unproven causal relation, Harrison's online lists rifampicin as cause of IN [5]. We present a patient with miliary tuberculosis, who developed IN during rifampicin, yet spontaneously recovered. Our observation and recent literature suggest that in case of hematogenously disseminated tuberculosis, a paradoxical response (PR) should be added to the list of possible causes of IN.

## Case presentation

**Figure 1:**
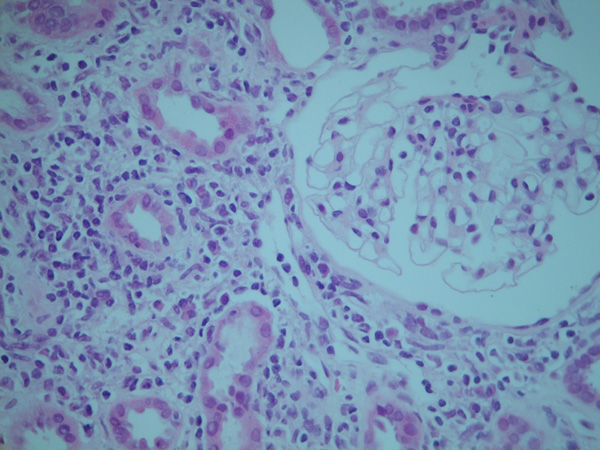
**Renal biopsy with a dense infiltrate in the interstitium, existing of lymphocytes, some plasma cells and a few eosinophils**. No signs of acid-fast bacilli or acute tubular necrosis.

An 18-year-old Ethiopian woman was presented with debilitating back pain, weight loss and high fever. A month earlier an abscess of her right buttock was surgically drained. Physical examination revealed an ill-looking young woman with a temperature of 40.1°C. The abdomen was painful at palpation and the liver slightly enlarged. The chest X-ray was compatible with diffuse pulmonary tuberculosis. A CT scan of the abdomen showed a psoas abscess on the left side. Laboratory data revealed: CRP 191 mg/l, Hb 6.0 mmol/l, lymphocyte count 0.8 × 10^9^/l, creatinine 93 micromol/l, blood urea nitrogen 3.8 mmol/l and serum albumin 20 g/l. Urinalysis showed no protein, cells or casts. HIV serology was negative. Acid-fast bacilli were found in the pus of the psoas abscess, so the diagnosis of hematogenously disseminated tuberculosis was established. Treatment with rifampicin, isoniazid, ethambutol and pyrazinamide was started. After 6 weeks on this four-drug regimen laboratory tests were repeated. Serum creatinine had risen to 272 micromol/l with a simultaneous rise of the lymphocyte count to 1.5 × 10^9^/l. Urinalysis showed 2+ protein, but no cells or casts. Protein excretion was 2.1 g/day. All four drugs were continued, albeit that the dosage of ethambutol was reduced with 50%. In the ninth week of treatment a renal biopsy was performed. Light microscopy revealed normal glomeruli but the interstitium showed a dense mononuclear infiltrate, existing of lymphocytes, some plasma cells and a few eosinophils. There were no signs of acute tubular necrosis. A Ziehl-Neelsen stain was negative for mycobacteria (Figure 1). On immunofluorescence: IgG was negative, IgA was positive in parts of some tubuli and IgM showed an atypical staining of the glomeruli. At the time of renal biopsy serum creatinine was 240 micromol/l and when the result of the biopsy became available it had decreased to 203 micromol/l. Because of this spontaneous recovery, all drugs were continued. Seven weeks after the renal biopsy, serum creatinine had returned to pre-treatment values. Nine months later treatment was stopped and the laboratory data were: Hb 8.0 mmol/l, creatinine 88 micromol/l, serum albumen 39 g/l and protein excretion 0.2 g/day.

## Discussion

The introduction of effective anti-tuberculosis treatment has been accompanied with acute renal failure. In the early fifties of last century nephrotoxicity of streptomycin, one of the first aminoglycosides, was causative. When rifampicin became available, acute tubular necrosis was described. Almost all of the patients had rifampicin-dependent antibodies against the I-antigen which is present on erythrocytes and also on tubular epithelial cells. This I-antigen therefore could be the target through which the drug leads to acute tubular necrosis [1]. In the nineteen-seventies, case histories of acute IN were published. Rifampicin was given for 21 to 71 days and renal biopsies showed interstitial infiltrates. The drug was stopped and renal function recovered. Due to this temporal relationship, an immune-mediated or direct nephrotoxicity of rifampicin was postulated [2]-[4]. However; such temporal relationship could also be compatible with a PR.

Paradoxical enlargement of intracranial tuberculomas during anti-tuberculosis treatment were reported in the early nineties of last century and called PR [6]. Later, with the start of highly effective anti-retroviral therapy, PR became more common in HIV-positive patients with hematogenously disseminated tuberculosis during anti-tuberculosis treatment. Recently, even IN in such a patient has been reported. Two months after the start of the combination of anti-retroviral and anti-tuberculosis treatment, renal failure developed and renal biopsy revealed interstitial infiltrates. Assuming PR, rifabutin a rifamycin not negatively affecting the anti-retroviral therapy was continued and prednisone was added. Renal function improved within two weeks [7].

PR is defined as a transient worsening of pre-existing, often symptomless, lesions during anti-tuberculosis treatment. The etiology is unknown, although an interaction between the host's immune response and mycobacterial products has been suggested. PR occurs more frequently in HIV-positive patients. Risk factors for developing PR in HIV-negative patients are: anemia (Hb<6.8 mmol/l), low serum albumin (< 30 g/l) and a low lymphocyte count (< 0.8 × 10^9^/l). Furthermore, during PR a significant rise of the lymphocyte count (>; 0.3 × 10^9^/l) is seen [8]. Anti-tuberculosis treatment during a PR should be continued. Our patient had hematogenously disseminated tuberculosis and abovementioned phenomena. Our hypothesis is that during the hematogenous spread mycobacteria are trapped within the kidney and cause IN i.e. PR during anti-tuberculosis treatment. The fact that the patient received quadruple therapy for nine weeks, could explain the absence of mycobacteria in the renal biopsy.

Transient IN during rifampicin treatment in HIV-negative tuberculosis patients has been described before, but not recognized as PR. One patient recovered after withdrawal of pyrazinamide while rifampicin was continued. The authors postulated pyrazinamide as the cause of the nephritis [9]. Another patient developed IN while receiving triple therapy. Rifampicin and isoniazid were stopped without effect. When ethambutol was stopped as well, renal function improved. Then rifampicin and isoniazid were reintroduced without detrimental effect. Thus ethambutol was considered the culprit [10]. Both patients however, had hematogenously disseminated tuberculosis and anemia. So PR seems a more likely explanation. Unfortunately, the case histories of rifampicin-associated IN contain insufficient data to draw conclusions about a possible PR.

## Conclusion

HIV-negative tuberculosis patients with well-defined risk factors may develop IN during anti-tuberculosis therapy. This nephritis should be considered a PR, thus anti-tuberculosis drugs could be continued and corticosteroids eventually added.

## List of abbreviations

IN: Interstitial nephritis; PR: paradoxical response; CT: Computed tomography; CRP: C-reactive protein.

## Consent

Written informed consent was obtained from the patient for publication of this case report and accompanying images. A copy of the written consent is available for review by the Editor-in-Chief of this journal.

## Competing interests

The authors declare that they have no competing interests.

## Authors' contributions

JvdM reviewed the case and relevant literature and prepared the manuscript. GJ reviewed and edited the manuscript. PW performed the histological examination. All authors read and approved the final manuscript.

## References

[B1] De Vriese AS Robbrecht DL Vanholder RC Vogelaers DP Lameire NH Rifampicin-associated acute renal failure: pathophysiologic, immunologic, and clinical features Am J Kidney Dis 1998 31 108 115 10.1053/ajkd.1998.v31.pm9428460 9428460

[B2] Gabow PA Lacher JW Neff TA Tubulointerstitial and glomerular nephritis associated with rifampin. Report of a case JAMA 1976 235 2517 2518 10.1001/jama.235.23.2517 946666

[B3] Neugarten J Gallo GR Baldwin DS Rifampin-induced nephrotic syndrome and acute interstitial nephritis Am J Nephrol 1983 3 38 42 10.1159/000166685 6837651

[B4] Power DA Russell G Smith FW Simpson JG MacLeod AM Friend JA Catto GR Acute renal failure due to continuous rifampicin Clin Nephrol 1983 20 155 159 6627764

[B5] Yu ASL Brenner BM2874673

[B6] Van Bommel EFH Stiegelis WF Schermers HP Paradoxical response of intracranial tuberculomas during chemotherapy: an immunologic phenomenon? Neth J Med 1991 38 126 130 1881499

[B7] Jehle AW Khann N Sigle JP Glatz-Krieger K Battegay M Steiger J Dickenmann, Hirsch HH Acute renal failure on immune reconstitution in an HIV-positive patient with miliary tuberculosis Clin Infect Dis 2004 38 32 35 10.1086/381441 14765361

[B8] Cheng SL Wang HC Yang PC Paradoxical response during anti-tuberculosis treatment in HIV-negative patients with pulmonary tuberculosis Int J Tuberc Lung Dis 2007 11 1290 1295 18034948

[B9] Sanwikarja S Kauffmann RH te Velde J Serlie J Tubulointerstitial nephritis associated with pyrazinamide Neth J Med 1989 34 40 46 2915734

[B10] Garcia-Martin F Mampaso F de Arriba G Moldenhauer F Martin-Escobar E Saiz F Acute interstitial nephritis induced by ethambutol Nephron 1991 59 679 680 10.1159/000186675 1766519

